# Neutrophil extracellular traps in cancer: From mechanisms to treatments

**DOI:** 10.1002/ctm2.70368

**Published:** 2025-06-13

**Authors:** Yifan Wang, Kangjie Yang, Juan Li, Chuanxin Wang, Peilong Li, Lutao Du

**Affiliations:** ^1^ Department of Clinical Laboratory Qilu Hospital of Shandong University, Shandong Provincial Key Laboratory of Innovation Technology in Laboratory Medicine Jinan PR China; ^2^ Department of Clinical Laboratory The Second Hospital of Shandong University Jinan PR China; ^3^ Shandong Provincial Clinical Medicine Research Center for Clinical Laboratory Jinan PR China

**Keywords:** biomarkers, cancer, neutrophil extracellular traps (NETs), therapeutic target

## Abstract

Neutrophil extracellular traps (NETs) are reticular ultrastructures released by activated neutrophils. As the reaction products of neutrophils, NETs have been identified as crucial effectors in pathogen defence and autoimmune diseases. Recently, increasing evidence suggest that this process also occurs in cancer. The formation and clearance of NETs are dynamically influenced by the tumour microenvironment, while NETs reciprocally play a dual role in either promoting or inhibiting tumour progression through their DNA scaffold, proteases and other granule‐derived proteins. Given the interplay between NETs and tumours, active exploration is currently underway to harness their potential as tumour biomarkers and therapeutic targets. Here, we delve into the biochemical and immunological mechanisms underlying NETs formation within the tumour microenvironment, along with recent advances elucidating their multifaceted roles in tumourigenesis, metastasis and tumour‐associated co‐morbidities. Furthermore, we present emerging strategies for NETs‐based tumour diagnostic approaches and therapeutics, with a special focus on the challenging questions that need to be answered within this field.

**Key points:**

- The formation and clearance of NETs are dynamically influenced by the tumor microenvironment.
- NETs are engaged in tumorigenesis, formation, metastatic spread, and cancer‐associated co‐morbidities.
- NETs‐based tumor biomarkers and therapeutic strategies warrant significant attention.

## Graphical abstract

Neutrophil extracellular traps are intricately linked to the tumor microenvironment and play a crucial role in tumorigenesis, metastasis, and related complications. Consequently, NETs‐based tumor biomarkers and therapeutic strategies warrant significant attention.

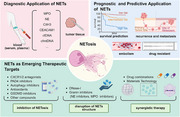

## 1 ADVENT OF NETOSIS

**FIGURE 1:**
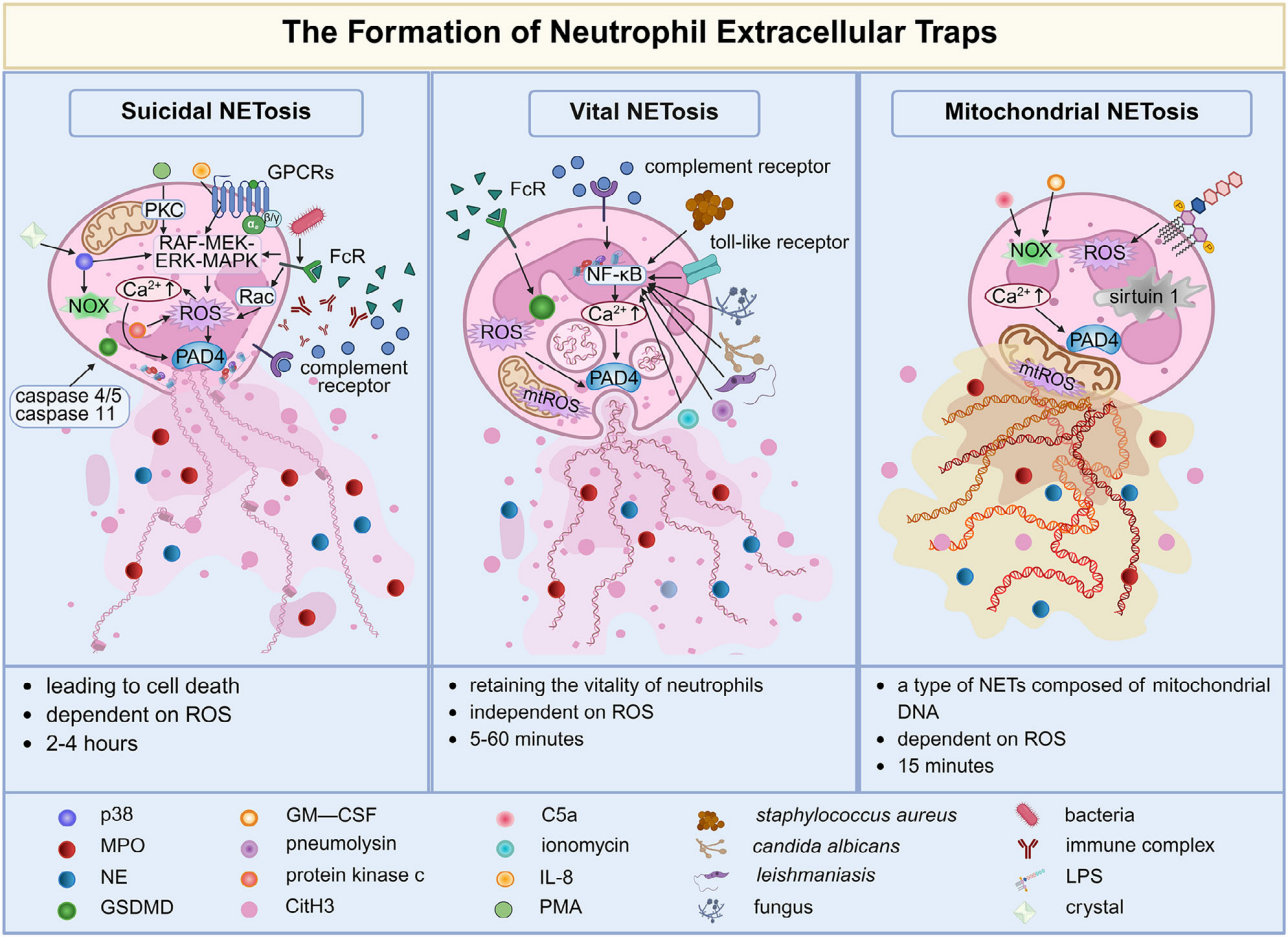
Schematic representation of neutrophil extracellular traps (NETs) formation. Three mechanisms of NETs formation (NETosis) have been described: suicidal NETosis, vital NETosis and mitochondrial NETosis, which differ in their stimuli, morphodynamics and biological mechanisms. Among them, suicidal NETosis, as the first mechanism to be described, activates many signalling pathways after being stimulated by bacteria, chemicals and immune complexes, leading to a variety of morphological changes in neutrophils, including nuclear structure changes, disaggregated chromatin, increased permeability of nuclear and granular membranes and the release of DNA and particles into the extracellular space. These can be detected several hours after neutrophils are activated. In contrast to suicidal NETosis, vital NETosis is Nox‐independent and does not require ROS formation. Moreover, neutrophils of this mechanistic type can survive after activation, while the nucleus, membrane and plasma membrane remain intact and maintain chemotactic and phagocytotic activities. Particulate matter released through vesicles can be detected minutes after cell activation. Mitochondrial NETosis forms NETs made of mitochondrial DNA rather than nuclear DNA, similar to suicidal NETosis, which also relies on ROS but does not lead to membrane rupture and cell death and is formed in a short time. C5a, complement component 5a; CitH3, citrullinated histone H3; ERK, extracellular regulated protein kinases; FcR, Fc receptor; GM‐CSF, granulocyte/macrophage colony‐stimulating factor; GPCRs, G protein‐coupled receptors; GSDMD, gasdermin D; LPS, lipopolysaccharide; MAPK, mitogen‐activated protein kinase; MEK, mitogen‐activated extracellular signal‐regulated kinase; MPO, myeloperoxidase; NE, neutrophil elastase; NOX, nicotinamide adenine dinucleotide phosphate oxidase; PAD4, protein arginine deiminase type IV; PMA, phorbol 12‐myristate 13‐acetate; Rac, Ras‐related C3 botulinum toxin substrate; RAF, rapid acceleration fibrosarcoma; ROS, reactive oxygen species. Image created with BioRender.com with permission.

Upon recruitment from circulation to sites of infection, neutrophils undergo activation by multiple stimulus and employ three strategies—phagocytosis, degranulation and neutrophil extracellular traps (NETs)—to effectively eliminate pathogenic threats.^1^ NETs are formed through a process known as ‘NETosis’, in which activated neutrophils release decondensed chromatin along with cytoplasmic components and granule‐derived proteins. Early in 2004, Arturo Zychlinsky's group first characterised the structure and composition of NETs.^1^ They discovered that upon activation by C–X–C motif chemokine ligand 8/interleukin‐8 (CXCL8/IL‐8), phorbol myristate acetate (PMA) or lipopolysaccharide (LPS), neutrophils actively released their decondensed chromatin and granule‐derived proteins such as neutrophil elastase (NE), myeloperoxidase (MPO), cathepsin G, lactoferrin and gelatinase. These components collectively formed extracellular web‐like fibres that effectively bound to and killed bacteria. Over the past two decades,^2^ our knowledge about the well‐orchestrated cellular process for NETs formation (NETosis) has significantly expanded. To date, the formation of NETs has been found to encompass multiple subtypes. In addition to classic suicidal NETosis, vital NETosis and mitochondrial NETosis, recent studies have identified derivative types such as pyroptosis‐related NETosis,^3^ autophagy‐related NETosis,^4^ endogenous‐stimuli‐induced NETosis,^5^ transcellular NETosis triggered by neutrophil–other‐cell interactions.^6^ Since the classification basis involves various dimensions including the source of stimulation, morphodynamic characteristics and molecular mechanisms and so forth, the criteria for NETosis subtypes remain unstandardised. Currently, the academic community commonly categorises NETosis into three primary types: suicidal, vital and mitochondrial. Herein, we emphasise these main types and discuss their distinct features (Figure 1).

### 1.1 Suicidal NETosis

Suicidal NETosis entails the release of NETs accompanied by lytic neutrophil death, through sequential cellular events. The cascade processes are frequently detected within 2–4 h and are reliant on the presence of reactive oxygen species (ROS). Concretely, suicidal NETosis is initiated upon neutrophil activation in response to multiple stimuli (PMA, CXCL8/IL‐8, immune complex, microorganisms, crystals, etc.). Neutrophil activation is triggered by various stimuli, including PMA, CXCL8/IL‐8, immune complexes and microorganisms. These stimuli are sensed by surface receptors, such as G‐protein‐coupled receptors, Fcγ receptors,^7^ and complement receptors.^8^ This receptor engagement leads to the release of calcium ions from the endoplasmic reticulum.^9^ The calcium influx further activates downstream kinase‐mediated signal transduction. For instance,^5^, ^10^, ^11^, ^12^ activation of protein kinase C, p38, mitogen‐activated protein kinase (MAPK) and Rac signalling induce the generation of ROS by the nicotinamide adenine dinucleotide phosphate (NADPH) oxidase (NOX) or mitochondrial respiration. High levels of ROS induce the release of NE and MPO from azurophil granules into the nucleus, where they promote chromatin decondensation through proteolytic histone cleavage and charge neutralisation, respectively.^13^, ^14^ Simultaneously, ROS, in synergy with intracellular calcium, activates protein arginine deiminase type IV (PAD4) to convert arginine to citrulline on histones, leading to chromatin decondensation.^9^, ^14^ Afterwards, decondensated chromatin disperses extensively within the cytoplasm, where it becomes intermingled with cytoplasmic and granule proteins. During the later stage, cytoplasmic NE binds to and degrades the cytoskeleton to block the phagocytic pathway.^13^ Caspases 4/5 or caspase 11 are also involved in suicidal NETosis by inducing the porin gasdermin D (GSDMD), which subsequently triggers plasma membrane rupture.^15^ Recently, a novel mechanism of NETosis has emerged, in which caspase 11 and GSDMD synergistically drive this process.^3^ It involves nuclear permeability, chromatin relaxation and plasma membrane rupture; however, it occurs independently of MPO, NE and PAD4. Morphologically, it resembles suicidal NETosis but with a distinct mechanism response.^16^

### 1.2 Vital NETosis

Pilsczek et al. surprisingly observed the rapid release of NETs within 5–60 min upon exposure to S*taphylococcus aureus*, even in the absence of lytic cell death.^17^ Instead, these NETs‐released neutrophils retained unusual crawling and phagocytic behaviours, integrating with the widespread tissue NETs to collectively limit bacterial dissemination. Subsequent investigations have reported that *Candida albicans*,^18^ *leishmania* ^19^ and ionomycin^14^ also induce this vital NETosis. During vital NETosis, neutrophils undergo sequential events including nuclear envelope blebbing, and trafficking of vesicles carrying nuclear DNA towards the plasma membrane followed by fusion with it, ultimately delivering the nuclear DNA out of the cells without plasma membrane perforation. Concurrently, some cytoplasmic granules are also discharged into the extracellular space through fusion with the plasma membrane and subsequently associated with extracellular nuclear DNA to form NETs.^20^ The vital NETosis is mediated by the toll‐like receptor 2/4 and complement receptor system,^20^, ^21^ but not ROS.

### 1.3 Mitochondrial NETosis

Mitochondrial NETosis initially discovered in 2009,^22^ represents an alternative form of NETosis characterised by the release of mitochondrial DNA rather than nuclear DNA from neutrophils. After priming neutrophils with granulocyte/macrophage colony‐stimulating factor (GM‐CSF) and administering complement factor 5a/LPS stimulation, within approximately 15 min, up to 80% of neutrophils released mitochondrial DNA into the extracellular space in a ROS‐dependent manner. The expelled DNA is coated with granule proteins, but nuclear proteins such as histones have not been detected. Studies have reported that in addition to NADPH oxidase, mitochondrial complexes I and III serve as supplementary sources of ROS for mediating the bactericidal activities of neutrophils.^23^, ^24^ Additionally, sirtuin 1, a deacetylates transcription factor, promotes the opening of mitochondrial permeability transition pore channels for the externalisation process of mitochondrial NETs.^25^ And presently, it has also been demonstrated that conditioned media from anaplastic thyroid cells can selectively induce the release of mitochondrial NETs in a ROS‐dependent and cell death‐independent manner.^26^

## 2 MECHANISMS OF NETOSIS IN CANCER

**FIGURE 2:**
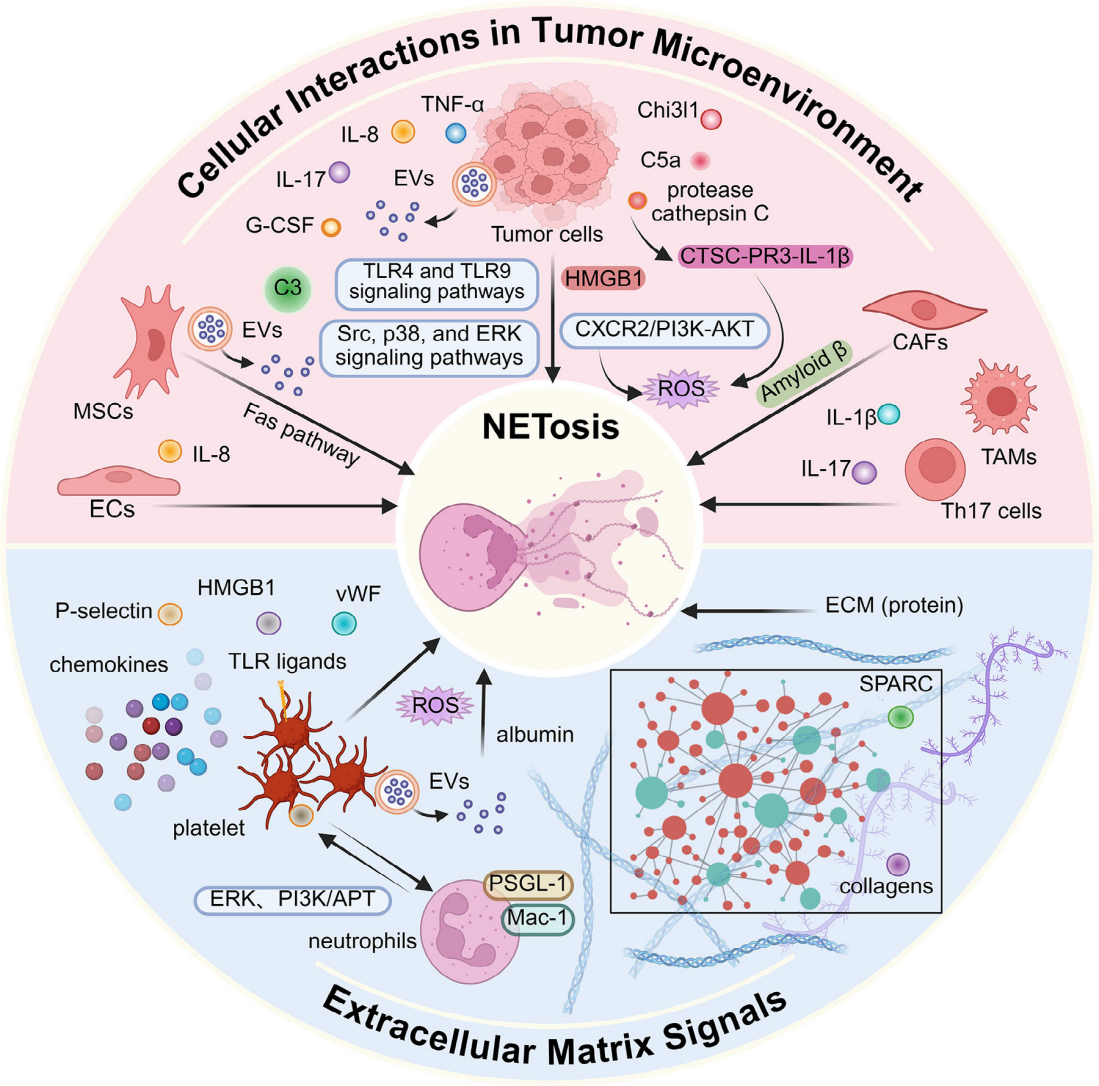
Mechanisms of neutrophil extracellular traps (NETs) formation in cancer. NETs formation is significantly influenced in the tumour microenvironment (TME), particularly through multiple cell interactions and signals from the extracellular matrix. Specifically, tumour cells can secrete cytokines, such as C5a, TNF‐α, IL‐8, IL‐17A and Chi3l1, which activate signalling pathways mediated by TLR4, TLR9, Src, p38, PI3K, AKT and ERK to induce NETosis. Endothelial cells and TH17 cells contribute to this process by secreting IL‐8 and IL‐17, respectively. Mesenchymal stromal cells regulate NETosis via the up‐regulation of complement C3 through the C3–C3aR axis or via vesicle‐mediated Fas pathways. Amyloid beta secreted by cancer‐associated fibroblasts also drives NETosis. The effects of complex protein components within the three‐dimensional non‐cellular framework of the ECM, such as SPARC and collagen, on NETosis are currently under investigation. Additionally, small molecules recruited to the TME during tumour development, such as platelets and albumin, influence NETosis by promoting ROS accumulation through distinct pathways. AKT, Ras‐related C3 botulinum toxin substrate, Rac; APT, abnormal prothrombin; CAFs, cancer‐associated fibroblasts; Chi3l1, chitinase‐3‐like protein 1; CTSC, cathepsin C; CXCR2, C–X–C motif chemokine receptor 2; C5a, complement component 5a; C3, complement receptor 3; ECs, endothelial cells; ECM, extracellular matrix; ERK, extracellular regulated protein kinases; EVs, extracellular vesicles; Fas, apo‐1/CD95/TNFRSF6; HMGB1, high mobility group box‐1 protein; IL‐1β, interleukin‐1 β; IL‐17, interleukin‐17; Mac‐1, macrophages‐1 antigen; MSCs, mesenchymal stromal cells; PI3K, phosphatidylinositol‐3‐kinase; PR3, proteinase 3; PSGL‐1, P‐selectin glycoprotein ligand‐1; ROS, reactive oxygen species; SPARC, secreted protein, acidic and rich in cysteine; SRC, proto‐oncogene tyrosine‐protein kinase Src; TAM, tumour‐associated macrophage; Th17 cells, helper T cell 17; TNF‐α, tumour necrosis factor‐α; vWF, von Willebrand factor. Image created with BioRender.com with permission.

Following the initial recognition of the significance of NETs in pathogen defence, subsequent studies have revealed their presence in non‐infectious inflammatory conditions, including cancer.^2^, ^27^ This heightened susceptibility towards NETosis is consistently observed across multiple tumour models, suggesting that cancer may systemically induce an increase in NETosis (Figure 2). The tumour microenvironment (TME) is a complex ecosystem surrounding tumour cells, which consists of a variety of cells, extracellular matrix, blood vessels and signalling molecules.^28^ Recently, these components have been found to trigger the formation of NETs via distinct pathways. This section will explore the specific mechanisms by which cancer promotes NETosis, focussing on the roles of cellular components and the extracellular matrix.

### 2.1 Cellular populations in TME trigger NETosis

#### 2.1.1 Tumour cells trigger NETosis

The neutrophil lifecycle and its effector functions can be influenced by tumour cells. For instance, tumour cells express inflammatory mediators and chemokines (e.g., G‐CSF, tumour necrosis factor‐alpha [TNF‐α], CXCL8/IL‐8 and IL‐17) to promote emergency granulopoiesis and tumour infiltration.^29^, ^30^, ^31^ These tumour‐infiltrating neutrophils undergo functional reprogramming towards a pro‐NETotic phenotype in the TME. Cytokines closely associated with tumour cells, such as complement factor 5a (C5a), TNF‐α, CXCL8/IL‐8, IL‐17A, GM‐CSF,^32^, ^33^ and chitinase‐3‐like protein 1 (Chi3l1), have been reported to trigger NETosis.^29^, ^34^ In diffuse large B‐cell lymphoma, the interactions between CXCL8/IL‐8 and the neutrophil receptor C–X–C motif chemokine receptor 2 (CXCR2) not only trigger NETosis through the proto‐oncogene tyrosine‐protein kinase Src (Src), p38 MAPK (p38) and extracellular regulated protein kinases (ERK) signalling pathways but also contribute to tumour progression.^35^ The up‐regulated release of CXCL8/IL‐8 and glutamate in non‐massive high‐grade plasmacytoid ovarian cancer is associated with the NETosis, as well as the hypoxic stress in tumour cells.^36^ Under stress conditions, chemokines (e.g., C5a) synergistically mediate PAD4 activation to trigger NETosis through TLR4 and TLR9 signalling pathways along with high mobility group box‐1 (HMGB1).^37^ In the advanced stage of tumour progression, necrosis caused by tumour cells tends to result in the release of damage‐associated molecular patterns, including mitochondrial DNA, which may trigger neutrophils to form NETs through the TLR9‐mediated signalling pathway.^38^ Additionally, tumour cells have also been found to secret protease cathepsin C, which stimulates ROS production via the cathepsin C‐proteinase 3‐interleukin‐1β (CTSC‐PR3‐IL‐1β) to support NETosis in breast cancer (BRCA).^39^ Except for releasing soluble factors, tumour cells secrete extracellular vesicles (EVs) as novel mediators for intercellular communication to modulate the functions of stromal cell and neutrophils. Guimarães‐Bastos et al. have demonstrated that treatment with human melanoma cells‐derived EVs facilitated neutrophil chemotaxis through CXCR2/phosphatidylinositol 3‐kinase (PI3K)‐serine/threonine kinase B (AKT) axis and increased ROS production to promote NETosis with poor elastase activity.^40^ In this process, EVs drive tumour‐infiltrating neutrophils to a pro‐tumour/N2 polarisation without phagocytic activity, ultimately contributing to tumour progression. In the colorectal cancer (CRC) study, EVs could transfer mutant V‐Ki‐ras2 Kirsten ratsarcoma viral oncogene homolog (KRAS) from tumour cells to recipient cells and induce CXCL8/IL‐8 production to induce neutrophil recruitment and NETosis.^41^ Furthermore, tumour cells‐derived EVs can also induce G‐CSF to trigger the formation of NETs which serve as a scaffold for pro‐coagulant exosomes involved in the establishment of cancer‐associated thrombus.^42^

#### 2.1.2 Stromal cells and infiltrating immune cells trigger NETosis

In addition to tumour cells, the TME also encompasses a complex network of non‐cancerous stroma.^43^ This stroma contains a diverse cellular population, including endothelial cells (ECs), mesenchymal stromal cells (MSCs) and cancer‐associated fibroblasts (CAFs), as well as a series of immune cells. Li et al.^44^ reported the interplay between ECs and neutrophil‐induced NETs in lung cancer (LC). Extracellular RNAs from lung tumour cells stimulated the production of inflammatory cytokine IL‐1β in lung ECs. This led to a decrease in the expression of vascular cell adhesion molecule‐1. These changes facilitated the recruitment of neutrophils to the lung and the initiation of NETosis. Similarly, activated ECs have also been reported to mediate the release of HMGB1 to induce NETosis.^45^ Furthermore, recent investigations have revealed that under chronic stress conditions, pulmonary ECs release increased levels of acetylcholine, which in turn enhances NETosis in aggregated neutrophils within the lung by activating muscarinic acetylcholine receptors.^46^ MSCs also have recently been reported to play dual roles in NETosis.^47^, ^48^, ^49^ In BRCA lung metastasis, MSCs could up‐regulate complement C3 to trigger NETosis through the C3–C3aR axis and promote lung metastasis of tumour cells in a STAT6‐dependent manner.^49^ On the other hand, EVs derived from MSCs inhibit NETosis or shift it towards apoptosis by activating the apoV‐Fas ligand‐mediated Fas pathway.^47^, ^48^ CAFs, another stromal population within the TME, have been found to secrete amyloid β to drive the formation of tumour‐associated NETs through CD11 antigen‐like family member B (CD11b) in a ROS‐dependent manner, both locally in the tumour and systemically in the blood and bone marrow.^27^ Interestingly, IL‐17, primarily secreted by T helper 17 (TH17) cells, has been reported to recruit neutrophils and promote NETosis, thus mediating immunosuppression by excluding cytotoxic CD8^+^T cells from tumours.^29^ Notably, the formation of IL‐17‐induced NETs required the involvement of factors released by tumour cells. It has been observed that NETs, in turn, prime macrophages for IL‐1β release, activating TH17 cells that amplify immune cell recruitment during sterile inflammation.

### 2.2 ECM signals trigger NETosis

The ECM is a three‐dimensional and non‐cellular framework that is essential for regulating tissue homeostasis including tumour progression, and consists of approximately 300 proteins, such as collagens.^50^ Sangaletti et al. propose that SPARC (cysteine‐rich secreted protein) deficiency, which is associated with impaired collagen assembly and defective ECM inhibitory signalling, can alter the ECM microenvironment. This alteration promotes spontaneous NETosis in vitro, leading to NF‐κB activation. However, the precise underlying mechanism requires further investigation.^51^ Additionally, collagen mediates CXCL5 production via the DDR1/PKCθ/SYK/NF‐κB signalling cascade, indirectly promoting neutrophil infiltration and NETosis.^52^ Type 1 collagen (Col1) in the extracellular matrix binds to discoidin domain receptor 1 (DDR1), activating the NF‐κB pathway to up‐regulate CXCL8 and subsequently trigger NETosis.^53^ Despite these findings, the crosstalk between the ECM and NETs within the TME remains poorly understood, necessitating additional research. Interestingly, experimental data in patients with essential hypertension have shown that NETs also expose a highly functional tissue factor capable of promoting endothelial collagen synthesis, potentially establishing a feedback loop between ECM remodelling and NETosis.^54^

### 2.3 Other signals trigger NETosis

Certain biomolecules, although not inherent constituents of the TME, can be recruited into the TME during tumour progression. Platelets, traditionally viewed as key players in hemostasis and thrombosis, have recently garnered attention due to their functional significance in tumour‐associated NETosis. This perspective is supported by the findings of Razak et al. who reported that platelets, when primed with conditioned medium from pancreatic cancer cells, induce the NETosis^55^ and similar observations pertain to CRC. Apart from platelets, plasma redox imbalance induced by albumin oxidation can also trigger NETosis via the accumulation of ROS in neutrophils. This promotes the colonisation of circulating tumour cells in pulmonary metastases.^56^

The microbiota, as a critical pathogenic factor for tumours, particularly those originating from the digestive tract, displays ecological imbalance in the TME and modulates NETosis through diverse mechanisms. Specifically, the oncogenic *Clostridium perfringens* induces NETs formation in CRC via the TLR4‐ROS signalling pathway and NOD‐like receptor (NOD1/2)‐dependent signalling.^57^ Additionally, in patients with hypertriglyceridemic pancreatitis, the decreased abundance of *Mycobacterium hominis* within the gut microbiome elicits IL‐17 release. Subsequently, this process activates the NF‐κB and IL‐17 signalling pathways to promote NETs formation.^58^ Recent studies have shown that the intratumoural microbiota can regulate tumour cell physiology and immune responses through various signalling pathways,^59^ highlighting the need for further research to elucidate the potential interplay between the intratumoural microbiota and NETs formation.

### 2.4 The influence of tumour‐associated neutrophils in NETosis

The heterogeneous effects of tumour‐associated neutrophils (TANs) in TME should also be acknowledged when investigating the triggers of NETosis.^60^ Analogous to the classification of tumour‐associated macrophages subtypes,^61^ TANs are primarily categorised into an anti‐tumour N1 phenotype and a pro‐tumour N2 phenotype.^62^ The N1 phenotype TAN is characterised as cytotoxic neutrophils with hypersegmented nuclei, marked by the up‐regulation of ICAM1, TNF‐α and CCL3.^63^ These cells inhibits tumour progression through the release of mediators such as ROS and TNF‐α, potentially reducing NETosis or enhancing anti‐tumour efficacy in NETs.^64^ Conversely, the N2 phenotype TAN, which promotes tumourigenesis, expresses various tumour‐promoting factors.^65^ Given the predominance of these cytokines in the TME, the N2 phenotype may facilitate NETosis and synergistically contribute to tumourigenic effects alongside NETosis.^52^ Recently, increasing evidence has indicated that the conventional dichotomy fails to comprehensively encompass the multiple phenotypic of TANs. More distinct TANs subtypes have been identified in cancers^66^, ^67^, ^68^, ^69^ (Table 1). For example, four TANs subtypes have been found in non‐small cell LC,^63^ while five TANs subtypes have been identified in pancreatic cancer.^70^ Nevertheless, as of now, there is limited evidence revealing the differential effects of these novel subtypes during the NETs formation. Hence, exploring each TAN subtype's roles in NETs formation in cancer will be a key focus in the future.

**TABLE 1:** Classification of tumour‐associated neutrophils subtypes.

Classification	TAN subtype	Corresponding markers	Functions/feature	References
Dichotomy	N1 (Anti‐tumourigenic)	TNFα; CCL3; ICAM‐1	Inhibit tumour growth, metastasis and immune escape by promoting anti‐tumour immune response and secreting tumour suppressor factors.	^71^
	N2 (pro‐tumourigenic)	CCL2; CCL3; CCL4; CCL5; CCL8; CCL12; CXCL1; CXCL2; IL‐8/CXCL8; CXCL16; CCL17; MMP‐9; VEGF; Bv8; Arginase	Induce cell mutation and DNA damage, secretes cytokines to promote tumour occurrence, development and metastasis and participates in immunosuppression.	^63^, ^71^
Trichotomy	N1 TAN	CD15^high^; CD54^+^; CD86^+^; CD101^+^; CD117^+^; CD170^low^; HLA‐DR^+^	Exert anti‐tumour effect through multiple channels.	^68^
	N2 TAN	VEGF‐A; ALPL; MMP‐9; CXCR‐4; CD11b^+^; PD‐L1; CD170^high^	Play the role of promoting tumour through multiple ways	^68^
	N0 TAN	CD10^−^; CD11b^+^; CD66b^+^; CD84^+^; JAML; LOXI; CD117^+^	The state between N1 TAN and N2 TAN.	^68^
Inquartation	TAN‐1	IL1RN; RIPK2; CD44	Suppress inflammation.	^66^
	TAN‐2	HLA‐DRA; CD74; HLA‐DMB; HLA‐DRB1	It is characterised by immunogenic antigen presentation.	^66^
	TAN‐3	C15orf48; CCL3; CCL4; CSTB; LGALS3	It is an activated state of neutrophils.	^66^
	TAN‐4	RPS12; RPL3; RPN2; RPL23	It is highly malleable.	^66^
Quincle	TAN‐0	–	It has no distinctive features and is an intermediate state of tumour invasion.	^70^
	TAN‐1	VEGFA; PLAU; LGALS3; LDHA; BHLHE40	It is an end‐state, promotes tumour development, has high glycolytic activity and is associated with poor prognosis.	^70^
	TAN‐2	NLRP3; PDE4B; CD69; IL1RN; ADM	It promotes inflammation in the TME.	^70^
	TAN‐3	VNN2; SELL	It is a transition state between PMN and TAN.	^70^
	TAN‐4	IFIT1; IFIT2; IFIT3; ISG15; RSAD2	It is associated with innate immune responses that preferentially express interferon‐stimulated genes.	^70^
Sextuple	Neu_01_MMP8	–	–	^60^
	Neu_07_APOA2	–	–	^60^
	Neu_08_CD74	–	–	^60^
	Neu_09_IFIT1	CD274; PD‐L1	It inhibits cytotoxic CD8 T cells.	^60^
	Neu_10_SPP1	–	–	^60^
	Neu_11_CCL4	CCL3; CCL4	It secretes chemokines and recruits macrophages.	^60^

Abbreviations: ADM, adrenomedullin; ALPL, alkaline phosphatase, liver/bone/kidney; BHLHE40, basic helix–loop–helix family member E40; C15orf48, chromosome 15 open reading frame 48; CCL12, C–C motif chemokine ligand 12; CCL17, C–C motif chemokine ligand 17 (TARC); CCL2, C–C motif chemokine ligand 2; CCL3, C–C motif chemokine ligand 3; CCL4, C–C motif chemokine ligand 4; CCL5, C–C motif chemokine ligand 5; CCL8, C–C motif chemokine ligand 8; CD44, hyaluronic acid receptor; CD74, MHC class II invariant chain; CSTB, cystatin B; CXCL1, C–X–C motif chemokine ligand 1; CXCL16, C–X–C motif chemokine ligand 16; CXCL2, C–X–C motif chemokine ligand 2; CXCR‐4, C–X–C motif chemokine receptor 4; HLA‐DMB, human leukocyte antigen DM beta chain; HLA‐DR^+^, human leukocyte antigen DR; HLA‐DRA, human leukocyte antigen DR alpha chain; HLA‐DRB1, human leukocyte antigen DR beta 1 chain; ICAM‐1, intercellular cell adhesion molecule‐1; IFIT1, interferon‐induced protein with tetratricopeptide repeats 1; IFIT2, interferon‐induced protein with tetratricopeptide repeats 2; IFIT3, interferon‐induced protein with tetratricopeptide repeats 3; IL1RN, interleukin 1 receptor antagonist; IL‐8/CXCL8, interleukin‐8 (C–X–C motif chemokine ligand 8); ISG15, interferon‐stimulated gene 15; JAML, junctional adhesion molecule‐like; LDHA, lactate dehydrogenase A; LGALS3, galectin‐3; LGALS3, lectin, galactoside‐binding, soluble, 3; MMP‐9, matrix metalloproteinase‐9; Neu, neutrophil; NLRP3, NOD‐like receptor family pyrin domain containing 3; PDE4B, phosphodiesterase 4B; PD‐L1, programmed death‐ligand 1; PLAU, plasminogen activator, urokinase; RIPK2, receptor‐interacting serine/threonine‐protein kinase 2; RPL23, ribosomal protein L23; RPL3, ribosomal protein L3; RPN2, ribophorin II; RPS12, ribosomal protein S12; RSAD2, radical S‐adenosyl methionine domain containing 2; SELL, selectin L; TAN, tumour‐associated neutrophil**;** TNFα, tumour necrosis factor‐α; VEGF, vascular endothelial growth factor; VEGF‐A, vascular endothelial growth factor A; VNN2, vanin 2.

### 2.5 Environmental conditions influencing NETosis

NETosis is not only modulated by the interplay between neutrophil status and various stimulatory factors within the TME, but also governed by the inherent properties of the microenvironment itself. For example, hypoxia within gastric tumours can induce the translocation of HMGB1 from the cytoplasm to the nucleus, subsequently triggering NET release via the TLR4/p38 MAPK signalling pathway.^72^ Furthermore, tumours also generate a hypertonic microenvironment via abnormal angiogenesis and inflammatory exudation.^73^ A recent study has demonstrated that NETosis increases exponentially with increasing osmolarity, and this phenomenon is independent of the stimuli used to increase osmolarity.^74^ Collectively, whether neutrophils elicit NETs depends on the dynamic balance between their cellular status and specific cancer‐associated conditions. Further in‐depth investigation is warranted to elucidate the synergistic mechanisms of diverse microenvironmental stimuli and their impact on the heterogeneity of NETosis.

### 2.6 NETs disruption for removal

The normal clearance of NETs is essential for maintaining the balance between NET formation and degradation.^75^ Under physiological conditions, completing their task of resisting pathogen invasion, NETs are cleared through a coordinated process involving cellular and enzymatic mechanisms and are ultimately metabolised in the liver and excreted via the kidneys.^76^ A key player in this process is DNase, which degrades the DNA that constitutes the primary scaffold of NETs in blood and tissues. By effectively cleaving the DNA structure, DNase induces the disintegration of NETs. Additionally, phagocytic cells such as macrophages directly engulf and digest NETs.^77^, ^78^, ^79^ When enzyme‐mediated degradation or cellular phagocytosis is impaired, excessive NETs accumulation can occur, leading to tissue damage. Thus, the maintenance of enzymatic degradation and cytophagic homeostasis is critical for efficient NETs clearance.

## 3 ROLES OF NETS IN CANCER

**FIGURE 3:**
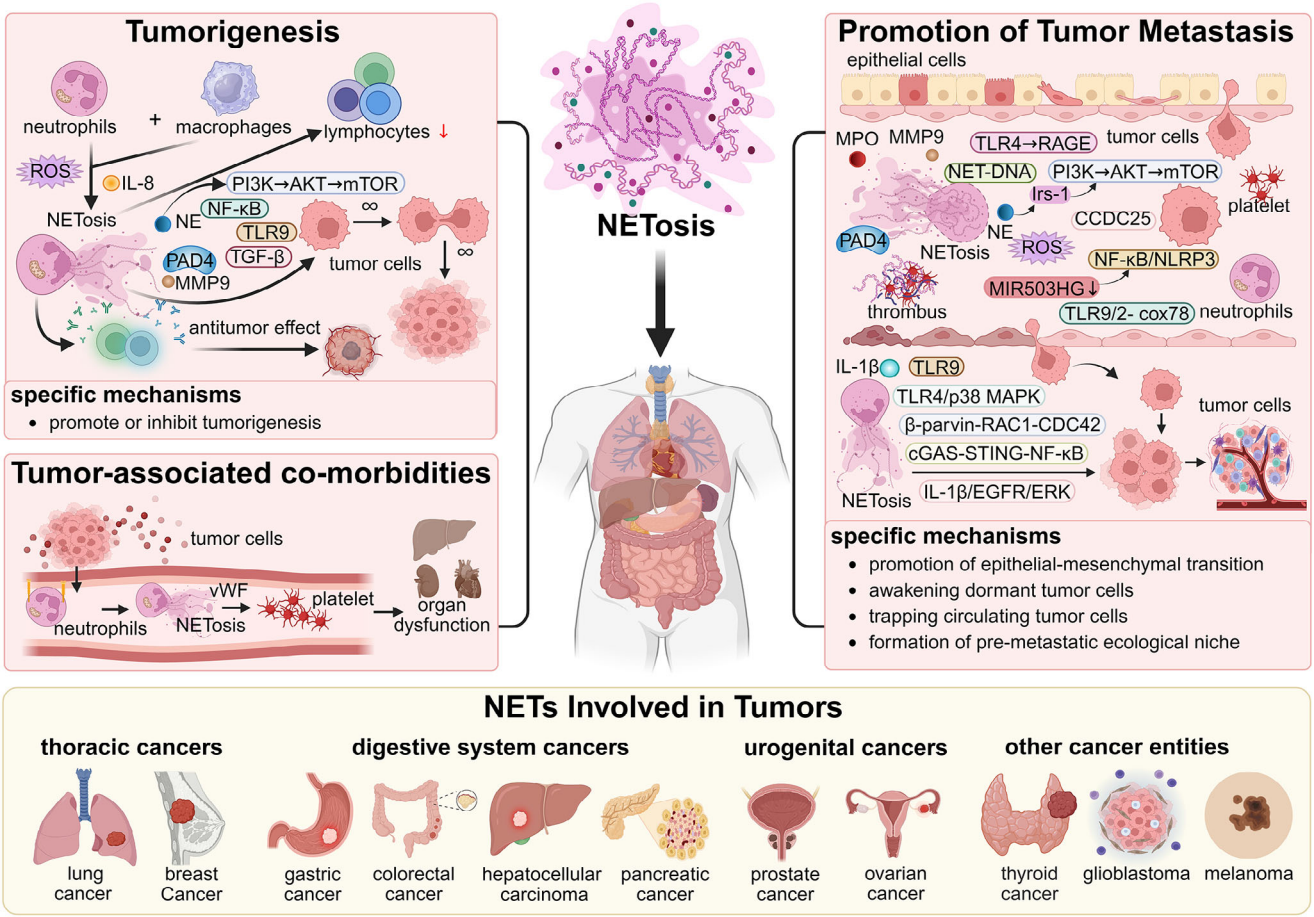
Roles of neutrophil extracellular trap (NETs) in cancer. NETs have been identified in a diverse range of human cancer types and are intricately associated with tumourigenesis, metastasis and tumour‐associated co‐morbidities. In addition to augmenting tumour cell proliferation, NETs can facilitate metastasis through distinct mechanisms, including promoting the epithelial–mesenchymal transition, awakening dormant tumour cells, trapping circulating tumour cells and establishing pre‐metastatic niches. Importantly, NETs likely contribute to the increased risk of thrombosis and even organ dysfunction observed in cancer patients. It is noteworthy that NETs may exhibit either pro‐ or anti‐tumour functions. AKT, protein kinase B; CCDC25, coiled‐coil domain containing 25; CDC42, cell division cycle 42; cGAS, cyclic GMP‐AMP synthase; cox78, cyclooxygenase 78; EGFR, epithelial growth factor receptor; ERK, extracellular regulated protein kinases; IL‐1β, interleukin‐1 beta; IL‐8, interleukin‐8; Irs‐1, insulin receptor substrate‐1; MEPK, mitogen‐Activated protein kinase; MIR503HG, MIR503 host gene; MMP9, matrix metallopeptidase 9; MPO, myeloperoxidase; mTOR, mammalian target of rapamycin; NE, neutrophil elastase; NF‐κB, nuclear factor kappa‐light‐chain‐enhancer of activated B cells; NLRP3, nucleotide‐binding oligomerisation domain, leucine‐rich repeat and pyrin domain‐containing 3; PAD4, protein arginine deiminase type IV; PI3K, phosphoinositide 3‐kinase; RAC, Ras‐related C3 botulinum toxin substrate; RAGE, receptor of advanced glycation endproducts; ROS, reactive oxygen species; STING, stimulator of interferon genes; TGF‐β, transforming growth factor beta; TLR‐4, Toll‐like receptor 4; TLR‐9, Toll‐like receptor 9; vWF, von Willebrand factor. Image created with BioRender.com with permission.

Acting as an extension of neutrophils, the involvement of NETs in a diverse range of pathologies is not surprising. The initial association between NETs and cancers was reported by Demers et al. in mice bearing mammary and lung tumours.^80^ Since then, experimental interventions targeting NETosis or NETs have gradually highlighted the causal role of NETs in cancers, including their involvement in carcinogenesis, formation, metastatic spread and cancer‐associated co‐morbidities (Table 2). Notably, depending on where NETs form and different TME statuses, NETs are likely to exhibit opposing pro‐tumour or anti‐tumour effects. Hence, we discuss examples illustrating how NETs contribute to tumour progression across diverse cancer types and stages (Figure 3).

**TABLE 2:** Summary of the roles of neutrophil extracellular traps (NETs) in cancers.

Cancer types	NETs affects cancers	Associated mechanisms/signalling pathways	References
Lung cancer	Promote lung cancer cell proliferation and migration and awaken dormant lung cancer cells.	PI3K/AKT pathway; NF‐κB pathway; NLR signalling pathway	^44^, ^56^, ^81^, ^82^, ^83^, ^84^, ^85^
Breast cancer	Promotes breast cancer cell proliferation and migration, especially lung metastases and facilitates immune escape from breast cancer.	NF‐κB pathway; PI3K/AKT/mTOR pathway; MAPK/ERK pathway; TGF‐β pathway; JAK/STAT pathway; VEGF pathway	^34^, ^42^, ^80^, ^86^, ^87^, ^88^, ^89^, ^90^, ^91^
Gastric cancer	Involvement in gastric carcinogenesis, progression, metastasis and immune escape.	AKT/mTOR pathway; TGF‐β signalling pathway; TLR pathway; VEGF pathway; JAK/STAT pathway; MAPK/ERK pathway	^92^, ^93^, ^94^, ^95^, ^96^, ^97^, ^98^, ^99^, ^100^
Colorectal cancer	Promote the growth, proliferation, distant metastasis and postoperative recurrence of colorectal cancer cells. But it also improves the anti‐tumour efficiency of adoptive natural killer cell therapy in mouse models of colon tumours.	TLR pathway	^57^, ^101^, ^102^, ^103^, ^104^, ^105^, ^106^, ^107^, ^108^
Hepatocellular carcinoma	Promote the inflammatory response of the body, inhibit the immunity of the body, make the tumour cells escape the immune surveillance and increase the potential of tumour metastasis. But it also Inhibit pancreatic cancer progression.	TLR4, TLR9 and TLR9/2‐COX78 signalling pathway; PI3K/AKT/Rac‐1 pathway; cGAS‐STING‐NF‐κB signalling pathway	^109^, ^110^, ^111^, ^112^, ^113^, ^114^, ^115^, ^116^, ^117^, ^118^, ^119^
Pancreatic cancer	Inhibit the immunity, enhance the invasion characteristics of pancreatic cancer and support the metastasis of pancreatic cancer.	IL‐1β/EGFR/ERK pathway	^120^, ^121^, ^122^
Prostate cancer	Promote the proliferation and metastasis of prostate tumour cells and induce the recovery of dormant tumour cells.	NF‐κB pathway; PI3K/AKT pathway; JAK/STAT pathway; MAPK/ERK pathway; TGF‐β signalling pathway	^123^, ^124^
Ovarian cancer	On the one hand, it promotes the occurrence, development and metastasis of ovarian cancer, and on the other hand, it improves the prognosis of patients by releasing S100A8 protein.	NF‐κB pathway; PI3K/AKT pathway; JAK/STAT pathway; MAPK/ERK pathway; TGF‐β signalling pathway	^125^, ^126^, ^127^, ^128^, ^129^, ^130^
Diffuse large B‐cell lymphoma	Promote tumour growth and lymph node spread.	TLR9 pathway; NF‐κB/STAT3/p38 pathways	^35^
Thyroid cancer	Promote the proliferation and cell cycle progression of thyroid cancer cells.	JAK2‐STAT3 pathway; NF‐κB pathway; MAPK/ERK pathway; TGF‐β signalling pathway	^131^, ^132^
Fibrosarcoma	Enhance the invasion ability of fibrosarcoma cells.	NF‐κB pathway; TGF‐β signalling pathway; NF‐κB pathway	^133^
Glioblastoma	Promote glioblastoma progression.	PI3K/AKT Pathway; MAPK/ERK pathway; NF‐κB pathway	^134^
Melanoma	On the one hand, it promotes melanoma growth and malignant metastasis, and on the other hand, it produces cytotoxic effects on tumour cells.	PI3K/AKT pathway; MAPK/ERK pathway; NF‐κB pathway	^135^, ^136^, ^137^, ^138^

Abbreviations: AKT, protein kinase B; cGAS, cyclic GMP‐AMP synthase; COX78, cyclooxygenase 78; EGFR, epidermal growth factor receptor; ERK, extracellular signal‐regulated kinase; IL‐1β, interleukin‐1β; JAK, Janus Kinase; JAK2, Janus Kinase 2; MAPK, mitogen‐activated protein kinase; mTOR, mammalian target of rapamycin; NF‐κB, nuclear factor kappa‐light‐chain‐enhancer of activated B cells; NLR, NOD‐like receptor; p38, p38 mitogen‐activated protein kinase; PI3K, phosphoinositide 3‐kinase; Rac‐1, Ras‐related C3 botulinum toxin substrate 1; STAT, signal transducer and activator of transcription; STAT3, signal transducer and activator of transcription 3; STING, stimulator of interferon genes; TGF‐β, transforming growth factor β; TLR, Toll‐like receptor; TLR4, Toll‐like receptor 4; TLR9, Toll‐like receptor 9; VEGF, vascular endothelial growth factor.

### 3.1 Thoracic cancer

Thoracic cancer mainly induces LC and BRCA. Substantial evidences have indicated the widespread presence of NETs within lung tissue, peripheral blood and sputum samples from patients with LC and underscored their significance in driving the progression of LC.^44^, ^56^ In mouse animal experimental models of LC, the NE signal derived from NETs or degranulation was observed to gain access to the inside of tumour cells and then degrade insulin receptor substrate‐1, thereby inducing a shift in the PI3K axis towards tumour cell proliferation.^81^, ^82^ In the lung adenocarcinoma (LUAD) mouse model, NETs have been demonstrated to promote the growth of LUAD by facilitating the degradation of SLC2A3 mRNA, inhibiting ferroptosis and CD8 (+) T cell activity. To elucidate the mechanisms underlying NETs‐mediated metastasis in LC, Wang et al. employed microarray analysis and discovered that NETs induced epithelial–mesenchymal transition (EMT) in tumour cells to acquire the motility and invasiveness through down‐regulating MIR503HG expression to activate the nuclear factor kappa‐B (NF‐κB)/NOD‐like receptor protein 3 inflammasome pathway.^82^ NE has the ability to hydrolyse a wide range of substrates, including collagen and lung surfactant, perhaps facilitating the spread and metastasis of early lesions. Interestingly, a recent study has reported that exposure to tobacco smoke induces sustained lung inflammation, leading to NETosis which promote the conversion of dormant tumour cells into highly aggressive metastases in mice.^84^

Metastasis is the major cause of BRCA‐related mortality. Apart from enhancing the metastatic potential of tumour cells directly^86^, NETs have been observed to act as physical scaffolds in the microvasculature of the liver and lung,^87^ capturing circulating tumour cells and facilitating their adhesion to tissue stroma for the formation of new metastatic foci.^87^ Dissociation of NETs with deoxyribonuclease I (DNase‐I) or inhibition of NETosis by PAD4 inhibitors effectively can diminish both the quantity and size of lung metastases in spontaneous and experimental metastasis murine models^88^, ^89^. Recent findings by Yang et al. propose an alternative role for NETs‐DNA in capturing tumour cells—it acts as a trap and functions as a chemotactic factor, attracting tumour cells.^90^ Their study reveals that NETs‐DNA can be sensed by coiled‐coil domain containing 25 (CCDC25) and subsequently initiates the β‐parvin‐RAC1‐CDC42 cascade, leading to cytoskeleton rearrangement and directional migration of BRCA cells.^90^ Notably, the presence of NETs have frequently been concomitant with venous thrombosis in advanced stages of mouse animal experimental models of BRCA.Several mechanisms have been proposed to explain this phenomenon, including platelet activation and a hypercoagulable state induced by the extracellular histone component of NETs. ^80^, ^139^, ^140^Therapy resistance is another contributor to mortality in BRCA. It has been reported that chemotherapy‐treated BRCA cells secrete IL‐1β, which triggers NETosis; this, in turn, induces a transforming growth factor‐β (TGFβ)‐dependent EMT in tumour cells and diminishes the therapeutic efficacy in mouse models of BRCA lung metastasis.^91^ Moreover, recent evidence links the formation of cytokine Chi3l1‐induced NETs to stromal restriction of CD8^+^T cells in triple‐negative BRCA, which partly accounts for unfavourable clinical outcomes and limited response to immune checkpoint blockade.^34^

### 3.2 Digestive system cancer

Gastric cancer (GC) is the fifth most common malignancy.^92^ Remarkable NETs formation has been observed in the plasma and tumour tissues of patients with GC, demonstrating a strong correlation with tumour stage and suggesting its potential involvement in tumour progression.^93^ Interestingly, the abundant deposition of NETs in TME accelerates tumour growth by promoting angiogenesis rather than directly enhancing the proliferative capacity of GC cells. Data from Yang et al. support this perspective; they found that blocking NETs with DNase‐I significantly inhibits tumour growth, which is associated with a decrease in microvessel density in murine subcutaneous tumour models.^94^ Mechanistically, upon stimulation of NETs, the NETs‐DNA receptor CCDC25 expressed on ECs, undergoes translocation to the cytoplasm and activates the AKT/mammalian target of rapamycin (mTOR) axis; this activation promotes proliferation and tubulation of ECs, thereby initiating neovascularisation and facilitating tumour growth. Peritoneal metastasis is widely recognised for its detrimental impact on the prognosis of patients diagnosed with GC. Xia et al. employed a murine model of postoperative abdominal infectious complications and observed that NETs in peripheral blood and ascites fluid promote extravasation and implantation of GC cells into the peritoneum and liver, facilitating their proliferation and metastasis through TGF‐β signalling.^95^ Recently, it has been demonstrated that a significant population of low‐density neutrophils is present in the peritoneal lavages of patients after radical gastrectomy. These low‐density neutrophils not only spontaneously generate abundant NETs without additional stimuli, but also facilitate the peritoneal metastasis of GC cells.^96^ Additionally, NETs have been found to play a crucial role in promoting GC metastasis by initiating the expression of COX‐2 through TLR2 ^97^ or via N4‐acetyl cytidine modification of SET and MYND domain‐containing protein 2 (SMYD2) mediated by N‐acetyltransferase 10 (NAT10).^98^ The abundant NETs deposited in GC can also damage umbilical vein ECs and trigger the release of Angiopoietin‐2 (ANGPT2) and tissue factors, leading to a hypercoagulable state.^99^ This hypercoagulable state not only increases the susceptibility to venous thromboembolism (VTE),^100^ but also promotes the entrapment of circulating tumour cells by NETs, thereby facilitating the formation of novel metastatic foci.

CRC is a prevalent malignancy of the digestive system characterised by strong heterogeneity, refractoriness to treatment and an unfavourable prognosis. Accumulating evidence has highlighted the contribution of NETs in the growth and dissemination of CRC cells in harsh microenvironments. NE released from NETs could activate TLR4 on CRC cells, leading to the up‐regulation of peroxisomes proliferator‐activated receptor gamma coactivator 1‐alpha and subsequent enhancement of mitochondrial biogenesis; this process provided extra energy for anabolic tumour growth. Of note, many clinical analyses have revealed a significant correlation between elevated levels of sera and pathological NETs markers and the higher risk of lymph node and liver metastasis for patients with CRC.^100^, ^101^, ^102^ In vivo, the study demonstrated that purified NETs induce filopodia formation and cell motility in CRC cell lines, which are accompanied by up‐regulation of mesenchymal markers (vimentin, fibronectin) and EMT‐promoting transcription factors (ZEB1, Slug), as well as down‐regulation of the epithelial markers E‐cadherin and epithelial cell adhesion molecule.^100^ In addition to inducing EMT in tumour cells, NETs can capture CRC cells and further enhance their malignancy via HMGB1‐ and CXCL8/IL‐8‐mediated TLR‐dependent pathways. Meanwhile, excessive HMGB1 and CXCL8/IL‐8 in turn activate neutrophils to release NETs, thereby establishing a positive feedback loop for hepatic micrometastases.^102^, ^103^ Interestingly, it has been discovered that neutrophils accumulate in the liver prior to the formation of new metastatic foci, concomitant with NETs formation.^104^ Currently, the mechanisms underlying NETs generation in the pre‐metastatic niche remain uncertain. It is possible that in CRC, gut microbiota may contribute to regulating NETs formation in the liver, due to its close correlation with PAMPs known to trigger NETosis.^105^ The study by Kong et al. supports this hypothesis. They discovered that *Fusobacterium nucleatum* indirectly accelerates CRC metastasis by regulating the formation of NETs in the pre‐metastatic niche induced by TME, thereby promoting angiogenesis and EMT.^57^ Surgical interventions for CRC are known to promote tumour recurrence and distant metastasis.^106^ Neutrophils, as major cellular responders to surgical stress, have recently been discovered to facilitate the colonisation of circulating tumour cells that have escaped from primary sites into target organs through NETs. In fact, increased postoperative NETs formation has been reported to be associated with a decreased disease‐free survival and an increased risk of complications for patients who undergo curative surgical interventions.^103^

Hepatocellular carcinoma (HCC) represents a common inflammation‐related carcinogenesis event, with over 90% of cases occurring in the context of virus infection, alcohol intake, metabolic syndrome and diabetes mellitus.^109^ S100A9, a PAMP induced by the hepatitis B virus, has been discovered to accelerate NETs formation and promote the growth of HCC.^110^ This pro‐tumour effect is mediated by the activation of TLR4 for advanced glycation end products (AGEs)‐ROS signalling. A study by Wang et al. reported that, in a choline‐deficient, high‐fat die + diethylnitrosamine mouse model and the stelic animal model, abundant NETs promote the differentiation of regulatory T‐cells (Tregs), thereby leading to immunosuppression in non‐alcoholic steatohepatitis and accelerating the development of non‐alcoholic steatohepatitis into HCC.^111^ NETs not only exert a direct impact on the occurrence of HCC,^87^, ^111^, ^112^ but also play a crucial role in mediating the migration, invasion and metastasis processes of HCC. By exploring the mechanism, it was found that NETs activated TLR4, TLR9 and TLR9/2‐COX78 signalling pathway,^89^ leading to the up‐regulation of COX2 and enhancing the toxic resistance and invasion ability of HCC cells.^112^, ^113^ Histone G in NETs has been found to promote HCC metastasis by down‐regulation of E‐calcineurin^141^; while MMP8 in NETs can activates TGF‐β 1 signal transduction through the lncRNA TP73‐AS1/miR‐539/MMP‐8 axis to induce polarisation of M2 macrophages in HCC and promote the further development of HCC.^115^ Furthermore, continuous accumulation of NETs‐DNA trigger HCC cell invasion through the cyclic GMP‐AMP synthase (cGAS)‐stimulator of interferon genes (STING)‐NF‐κB signalling pathway.^116^ Actually, several studies revealed that the deposition of NETs is most evident in the liver compared to other organs, such as the lung, bone and skin.^89^, ^117^ This phenomenon may be partially attributed to the lower blood shear forces in hepatic sinusoids, which makes NETs less susceptible to disruption. Moreover, NETs components can reboot NETosis during the self‐renewal process of blood vessels.^117^ Of note, these extensive NETs have also been observed in a murine hepatic ischemia/reperfusion injury model of localised surgical stress, which traps aggregated circulating tumour cells and eventually promotes postoperative tumour recurrence and metastasis.^118^, ^119^

Pancreatic cancer remains a global health‐care challenge due to its formidable mortality rate.^92^ The elevation of NETs in pancreatic cancer has been substantiated by studies, implicating their involvement in the proliferation, metastasis and resistance to immune checkpoint blockade of this malignancy.^120^, ^121^ NETs enhance the aggressive characteristics of pancreatic cancer, mechanistically by activating the IL‐1β/epidermal growth factor receptor (EGFR)/ERK pathway, interacting with receptors for RAGE and contributing to the loop of IL‐17 immunosuppression in a PADI4‐ and/or the receptor of advanced glycation endproducts (RAGE)‐dependent manner.^29^, ^122^, ^142^ Meanwhile, NETs also activate CAFs to promote liver micrometastasis in PDAC.^121^ Actually, the incidence of VTE varies across different cancer types, with BRCA demonstrating a lower occurrence while pancreatic cancer presenting a higher propensity for VTE. The presence of NETs in pancreatic cancer has been found to induce a prothrombotic state by activating platelets and releasing tissue factors.^55^, ^143^ The involvement of DNA and the receptors for RAGE is essential for initiating NETs‐mediated platelet aggregation.^143^ Furthermore, targeting this pathway with the NETs inhibitor chloroquine may reduce the risk of VTE.^143^

In summary, NETs play a critical role in regulating digestive system cancers through multiple mechanisms, such as promoting angiogenesis, inducing tumour metabolic reprogramming, trapping circulating tumour cells, promoting EMT and triggering prothrombotic state. However, the specific mechanisms by which NETs function in different tumour types vary significantly, likely due to differences in tissue origin, molecular characteristics and TME. Future research should focus more deeply on the functional distinctions and spatially specific regulation of NETs across various cancer types. Moreover, these discoveries offer new insights into potential therapeutic strategies for digestive system cancers, such as targeting the inhibition of NETs formation or modulating associated signalling pathways, which may pave the way for more precise treatment approaches.

### 3.3 Urogenital cancer

Recently, it has been discovered that NETs exert a significant impact on the progression of genitourinary system tumours by promoting inflammatory responses, inducing angiogenesis and facilitating tumour invasion. In prostate cancer, NETs promote the proliferation of the DU145 human prostate tumour cell line through up‐regulating CXCL8/IL‐8 expression and releasing PAD4.^123^ Moreover, NETs have been found to induce dormant tumour cells to awaken, thereby leading to the formation of lung metastases in a murine RapidCaP prostate cancer model.^84^ Additionally, MPO within NETs affects susceptibility to prostate cancer through genetic polymorphisms.^124^ The omentum is a common site of metastatic dissemination for ovarian cancer. Increased NETosis has been found in the omentum of patients with early‐stage ovarian cancer and in murine pre‐metastatic models.^125^, ^126^ Several studies reported that NETs can capture tumour cells at high levels within the omental wall before metastasis occurs in stage I and II ovarian cancer,^127^ thereby promoting metastasis to the greater omentum.^128^ This phenomenon has been positively correlated with reduced progression‐free survival in advanced epithelial ovarian cancer.^129^ While evidence suggests that NETs facilitate the occurrence and development of ovarian cancer, they also exert certain anti‐tumour effects. Specifically, NETs release S100A8 protein, leading to an increase in the S100A8/CRP ratio which has been positively associated with survival rate.^36^ This correlation has also been confirmed in patients with high‐grade serous ovarian cancer.^130^

In conclusion, NETs play dual roles in urogenital cancers, such as promoting tumourigenesis in prostate cancer while suppressing it in ovarian cancer. Future research should aim to elucidate the mechanisms underlying these dual functions during cancer progression and explore the regulatory balance by which NETs either promote or inhibit cancer development. This understanding will facilitate the optimisation of targeted therapeutic strategies.

### 3.4 Other cancer entities

The effects of NETosis on other solid tumours exhibit intricate and diverse characteristics. In diffuse large B‐cell lymphoma, NETs induce the up‐regulation of the TLR9 pathway in vivo^35^ and activate the NF‐κB, STAT3 and p38 pathways, thereby promoting tumour growth and lymph node dissemination. A similar process has been identified in thyroid cancer.^26^ The major biomarkers in NETs (dsDNA, nucleosomes, CitH3 and MPO‐DNA complexes) were positively associated with malignant progression of thyroid cancer.^26^ Anaplastic thyroid cancer (ATC) induces NETs on the CXCL‐8/IL‐8/ROS axis, while NETs promote ATC cell growth by maintaining its viability.^131^ In fibrosarcoma, elastase, cathepsin G and protease‐3 derived from NETs can enhance the invasion of HT1080 fibrosarcoma cells by activating matrix metalloproteinase‐2 and Membrane‐type 1 matrix metalloproteinase (MT1‐MMP).^133^ In glioblastoma, NETs regulate the HMGB1/RAGE/CXCL8/IL‐8 axis and expand the interaction between glioma progression and the TME.^134^ Moreover, the accumulation of NETs within TME promotes the growth of melanoma cells^144^ and also opens the endothelial barrier leading to an increase in melanoma cell endosmolar^135^ and NETs infiltration has also been found in malignant melanoma metastasis.^136^

However, NETs produce anticancer effects in addition to pro‐cancer effects on tumours, just as they were initially found to have anti‐infective effects. Studies have reported that an increase in the number of N1‐TAN in a mouse model of pancreatic cancer under melatonin treatment led to an increase in NETs producing anti‐tumour immunity, resulting in pancreatic cancer tumour suppression.^64^ Similarly, Src kinase‐associated phosphoprotein 1 (SKAP1)‐induced NETs significantly increased the over‐representation of natural in a mouse model of colon tumours. Anti‐tumour efficiency of killer cell therapy.^108^ In vitro studies have demonstrated that NETs exert cytotoxic effects on tumour cells by inhibiting the proliferation of non‐metastatic and metastatic melanoma cells through integrin‐mediated adhesion to limit migration and proliferation of cultured human melanoma cells.^137^ Furthermore, Bacillus Calmette–Guérin‐induced NETs have been shown to induce G0/G1 phase arrest and apoptosis of tumour cells in a dose‐ and time‐dependent manner, thereby inhibiting tumours.^145^ Therefore, the impact of NETs on a variety of tumours needs to be further explored.

## 4 DETECTION TECHNIQUES FOR NETS

### 4.1 Traditional NETs detection technology

Although NETs were discovered over two decades ago, there is still no universally accepted gold standard for their detection. Traditional methods mainly consist of immunofluorescence microscopy,^146^, ^147^ flow cytometry,^148^, ^149^ and ELISA‐based assays for detecting NETs‐DNA and related proteins. However, these methods have their limitations. Immunofluorescence microscopy is prone to operator bias and has low throughput.^150^ Flow cytometry indirectly detects NETs by assessing specific neutrophils infiltration, such as MPO‐positive neutrophils.^113^, ^151^ ELISA‐based assays also lack information on early time points during NETosis.^150^ As a result, many researchers have attempted to address these shortcomings by integrating multiple techniques or devising innovative approaches.^147^, ^152^, ^153^

### 4.2 Improved NETs detection technology

Focussed on NETs‐DNA, Brinkmann et al.^154^ quantified NETs in vitro by employing anti‐chromatin antibodies, correlated fluorescent signals with DNA‐binding dye signals, and automatically calculated the percentage of NETs based on the nucleus area and chromatin staining intensity. Singhal et al.^155^ developed a cellomics platform based on high‐content screening (HCS) imaging and HCS‐Cellomics algorithms, which used non‐permeable DNA staining to differentiate membrane‐permeable NETs‐DNA from other forms of cell death. The automated algorithm‐driven single‐cell analysis accurately detected NETs through the examination of nuclear morphological changes, increases in nuclear area and intensity alterations. This not only enables detailed temporal and longitudinal studies of NETosis but also provides valuable insights into the dynamic processes involved. Regarding NETs‐proteins, multiple immunofluorescence techniques were utilised, targeting CD15, MPO and citrullinated histone H3 (CitH3) as markers to quantify NET abundance in biopsy tissues.^156^ A novel method was devised for tandem labelling of the NETosis reporter gene 1, enabling specific detection of NETosis by activating the fluorescent signal exclusively in the presence of NE and histone G.^157^ Recently, Gavillet et al.^158^ reported a quantitative approach based on flow cytometry and immunological assays to identify and quantify NETs using antibodies against key NET components (specifically DNA, modified histones and granzymes). This method is applicable for detecting NETs induced both in vitro and in vivo in blood samples.

In order to enhance the accuracy of NETs detection, Coelho et al.^159^ proposed a framework for automatically identifying NETs based on NETs‐DNA and NETs‐proteins. This framework quantifies the NETs area in fluorescence microscope images by integrating cell surface area, DNA deformability and levels of NETs‐binding proteins. Considering the diversity of NETosis types, Zhao et al.^151^ developed a novel technique for distinguishing modes of NETosis using multi‐spectral imaging flow cytometry combined with image analysis. By leveraging multiple fluorescence images, including transmitted light, side scatter, cellular components and nuclear features extracted via software, this method facilitates quantitative analysis of both suicidal NETosis and vital NETosis. It offers the benefits of automation, precision and rapidity; however, capturing the advanced stages of NETosis with high accuracy remains challenging. Currently, emerging technologies such as artificial intelligence, mass spectrometry, nanotechnology and single‐cell sequencing are creating new opportunities for NETs detection, prompting scholars to continuously improve and refine detection methods.

## 5 CLINICAL APPLICATIONS OF NETS IN CANCER

**FIGURE 4:**
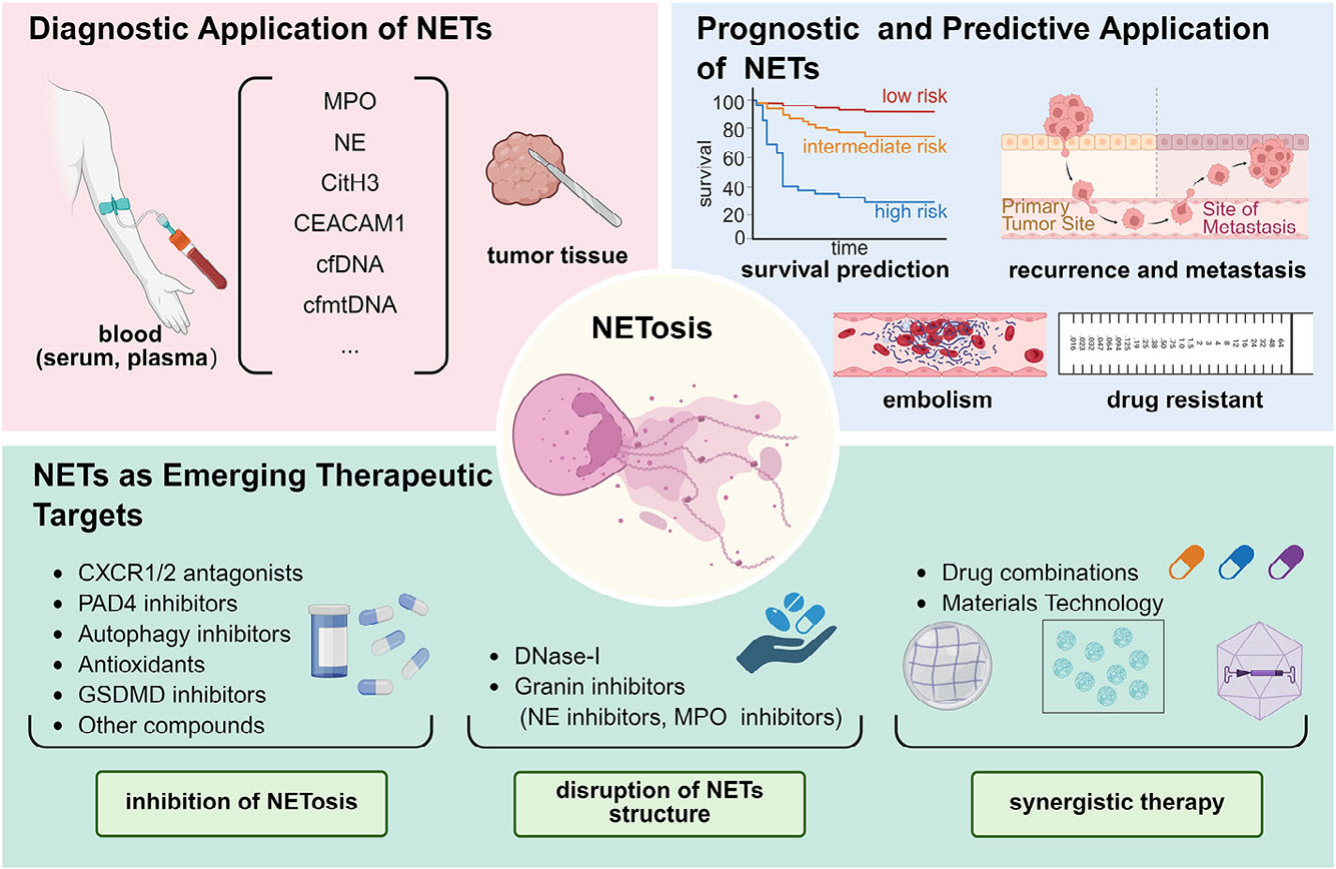
The clinical application of neutrophil extracellular traps (NETs) in cancer. The application of NETs in cancer clinical practice benefits from the progressive advancement of detection techniques, such as multi‐immunofluorescence, multi‐spectral imaging flow cytometry, flow cell‐omics platform and microfluidic devices. Aberrant levels of NETs markers have been widely observed in the blood and tissues of cancer patients, potentially aiding in cancer diagnosis, prognosis assessment and treatment efficacy evaluation. Furthermore, the therapeutic strategies for NETs primarily revolve around inhibition of NETs formation (NETosis) and disruption of already formed NETs. CEACAM1, carcinoembryonic antigen‐related cellular adhesion molecule1; cfDNA, circulating free DNA; cfmtDNA, circulating free mitochondrial DNA; CitH3, citrullinated histone H3; G‐CSF, granulocyte colony‐stimulating factor; GSDMD, gasdermin D; MPO, myeloperoxidase; NE, neutrophil elastase; PAD4, protein arginine deiminase Type IV. Image created with BioRender.com with permission.

Given the extensive involvement of NETs in tumour progression, hold great promise as emerging targets for tumour diagnosis and treatment. Currently, there is a growing interest in the clinical application of NETs within the field of oncology. Next, we discuss the detection technology, diagnostic value, efficacy evaluation and targeted therapy of NETs (Figure 4).

### 5.1 Diagnostic application of NETs

Recent evidence has demonstrated apart from their presence in tumour tissues, NETs undergo degradation into soluble nucleosomes, leading to the release of DNA and granule proteins fragments into peripheral blood.^160^ As the tumour burden increases, these circulating NETs markers levels gradually elevate and show a closer association with the clinical pathological characteristics of tumours, suggesting their significant potential as diagnostic biomarkers for tumour detection.^161^, ^162^ It has been suggested that the NE‐DNA complex had a superior diagnostic value compared to carcinoembryonic antigen (CEA) and carbohydrate antigen 19‐9 (CA19‐9) as serum biomarkers in GC.^161^ The plasma levels of three other NETs markers (MPO, NE and circulating DNA) were highly correlated and demonstrated significant discriminatory capacity in distinguishing metastatic CRC patients from healthy controls, both individually and collectively.^102^ Carcinoembryonic Ag cell adhesion molecule 1 (CEACAM1), expressed in the structural components of NETs, is considered a promising diagnostic marker for melanoma, as well as breast, pancreatic and bladder cancers.^163^, ^164^, ^165^, ^166^ Additional putative markers for NETs include cfDNA and cell‐free mitochondrial DNA (cfmtDNA). Ronchetti et al.^167^ observed a positive correlation between cfDNA and CitH3 content, while noting an inverse relationship between CitH3 and cfmtDNA, specifically evident in patients with endometrial cancer (EC) rather than healthy controls. They further demonstrated the potential of serum cfDNA, CitH3 and cfmtDNA levels as auxiliary tools for EC detection. However, it is important to note that the diagnostic utility of cfDNA and cfmtDNA should be interpreted cautiously, as they may not solely represent NETs but could also derive from dying or apoptotic cells.^168^, ^169^, ^170^

### 5.2 Prognostic and predictive application of NETs

In both experimental models and cancer patients, the deposition of NETs within tumour tissues exhibited a potential association with tumour progression.^90^, ^171^ Tumour‐infiltrating NETs, characterised by positive immunostaining with anti‐CitH3 and either anti‐MPO or anti‐CD15 antibodies, have been identified as an independent prognostic factor in patients undergoing radical resection for pancreatic and oesophageal cancer.^172^, ^173^, ^174^ In terms of predicting treatment efficacy, a study has reported tumour‐infiltrating NETs are negatively correlated with response to radiotherapy in muscle‐invasive bladder, and associated with poorer overall survival.^175^ Recently, accumulating evidence has highlighted the prognostic role of circulating NETs markers in tumour progression.^35^, ^85^ Yang et al. observed that plasma or serum MPO‐DNA complex levels were significantly elevated in BRCA patients who developed liver metastases compared to those who did not or those who developed metastases to other organs (e.g., lungs, bone and brain),^90^ and that their elevation was an independent variable that correlated with subsequent liver metastasis‐specificity rather than metastasis to other organs. Notably, the circulating MPO‐DNA complex has also demonstrated its potential in predicting the risk of intra‐abdominal infection, recurrence, liver metastasis and disease‐free survival for CRC patients following radical surgery.^104^ Elevated levels of circulating CitH3 or NE‐DNA are strongly associated with an unfavourable clinical outcome in patients with advanced active cancers, with approximately a two‐fold increased risk for short‐term mortality.^161^, ^167^ Apart from quantifying the circulating levels of NETs markers in serum or plasma, certain studies have assessed the release of NETs from purified neutrophils from peripheral blood. For instance, employing simple SYTOX staining of blood neutrophils following in vitro stimulation has shown promise as a potential prognostic tool for predicting the TNM stage in head and neck cancer.^176^ On the contrary, an increased proportion of NET‐producing CD16^high^CD62L^dim^ neutrophils in blood has been reported to be associated with improved survival rates owing to their anti‐tumour activity in suppressing proliferation, migration and inducing apoptosis for head and neck squamous cell carcinoma.^176^

Cancer‐associated thromboembolism significantly contributes to increased mortality and morbidity; however, the current approach for identifying high‐risk patients remains limited. A prospective study has demonstrated a constant association between CitH3 and VTE specifically in patients with pancreas and LC, while no such association was observed in individuals with brain and CRC.^177^ An increase of 100 ng/mL in plasma CitH3 levels was found to be associated with a 13% relative rise in cancer‐associated VTE risk, and elevated plasma levels of nucleosome and cfDNA levels, two putative NETs markers, were also observed to be linked to an increased risk of VTE, exclusively within the initial 3–6 months. In patients with pancreatic ductal adenocarcinoma and distal extrahepatic cholangiocarcinoma, calprotectin within NETs has shown promise as a predictor and monitor for VTE.^178^ Moreover, in glioma patients, a plasma‐based model incorporating MPO and cfDNA levels demonstrates superior predictive accuracy for early post‐surgical pulmonary embolism compared to the predictive efficacy of the Khorana score.^179^ Collectively, NETs markers can serve as predictive indicators for thromboembolism risk in cancer patients, thereby facilitating personalised thromboprophylaxis in a specific subset of high‐risk individuals.

### 5.3 NETs as emerging therapeutic targets

As discussed above, despite the current incompleteness in our understanding of the formation and functional mechanisms of NETs, NETs may exert pivotal roles in tumour initiation, progression and associated co‐morbidities. Therefore, emerging therapeutic strategies are focussed on inhibiting NETosis and (or) reducing already formed aberrant NETs (Table 3).

**TABLE 3:** Potential targets for interventions against neutrophil extracellular traps in cancers.

Therapeutic mechanism	Target	Compounds	Cancers	References
Inhibition of NETosis	CXCR1/2	Reparixin	Breast cancer, Lewis lung cancer, primary melanoma, colorectal cancer, HER‐2‐negative breast cancer	^180^, ^181^, ^182^, ^183^, ^184^
		SX‐682	Melanoma	^185^
		AZD5069	Pancreatic ductal adenocarcinoma, prostate cancer	^180^, ^183^, ^184^
		Navarixin	Advanced solid tumours	^186^
		CXCR2 inhibitor	Pancreatic ductal adenocarcinoma;	^187^
	IL‐8/CXCL2	IL‐8 antibody/ CXCR2 antibody	Diffuse large B‐cell lymphoma	^35^
		IL‐8 antibody	Glioma	^134^
	PAD4	YW3‐56	Nasopharynx cancer	^188^
		BMS‐P5	Multiple myeloma	^189^
		GSK484	Nasopharynx cancer, ovarian cancer, hepatocellular carcinoma, melanoma	^27^, ^190^, ^191^
		Cl‐amidine	Ovarian cancer, breast cancer, melanoma	^128^, ^192^
		Chloramidine	Pancreatic ductal adenocarcinoma	^193^
		ZD‐E−1 M	Breast cancer, lung cancer	^194^
		BB‐Cl‐amidine	Ovarian cancer	^190^
	PADI2	Icariin	Urothelial carcinoma	^195^
	Autophagy	Chloroquine	Pancreatic cancer	^196^
		Low molecular heparin	Colorectal cancer	^57^
	ROS	Kaempferol	Breast cancer	^197^
		pepducin	Pancreatic cancer	^187^
	Nicotinamide adenine dinucleotide phosphate (NADPH)	DPI	Colorectal cancer	^198^
	GSDMD	Ivermectin	Melanoma cancer	^144^
	thromboxane A2	Acetylsalicylic acid	Breast cancer	^199^
	HMGB1	Thromboregulin	Pancreatic cancer	^120^
		Antithrombin III	Endotoxemia	^200^
	TGF‐β	LY2157299	Gastric cancer	^201^
		SB525334	Pancreatic cancer	^202^
	CTSC	AZD7986	Breast cancer	^39^
	BCAM‐1	BCAM‐1 inhibitor	Pancreatic cancer	^203^
	IL‐17	Anti IL‐17 antibody	Lung cancer; pancreatic cancer	^29^, ^204^
	CEACAM1	CEACAM1 antibody	Colorectal cancer	^164^
	Integrin α5β1	ATN‐161	Colorectal cancer	^198^
	CXCL5	DDR1	Pancreatic ductal adenocarcinoma	^52^
	Other	PGE1	Pancreatic cancer	^205^
		KRAS mutation inhibitors	Colorectal cancer	^41^
Disruption of neutrophil extracellular traps (NETs) structure	DNA	DNase‐I/rhDNase I	Head and neck cancer, bladder cancer, breast cancer, colorectal cancer	^206^, ^207^, ^208^, ^209^, ^210^
	NE	Sivelestat	Prostatic cancer, colorectal cancer	^85^, ^100^, ^211^, ^212^
		ONO‐5046	Lung cancer	^81^
		Elafin	Breast cancer	^213^
Combined treatment	DNase I combined with PD‐1 antibody		Colorectal cancer	^214^
	Navarixin combined with aspirin or hydroxychloroquine		Hepatocellular carcinoma	^113^
	PAD4 inhibitor combined with PD‐1 inhibitor		Pancreatic tumour	^29^

Abbreviations: CXCR2, C–X–C motif chemokine receptor 1/2; G‐CSF, granulocyte colony‐stimulating factor; HMGB1, high mobility group box‐1 protein; NE, elastase; PAD4, protein arginine deiminase Type IV; ROS, reactive oxygen species; TGF‐β, transforming growth factor beta.

#### 5.3.1 Inhibition of NETosis

##### CXCR1/2 antagonists

One potential strategy for targeting NETs would involve interfering with their formation. Theoretically, this could be achieved by targeting the factors that are essential for NETosis, such as neutrophil activators, PAD4 and ROS generation. Compounds against CXCL8/IL‐8 and CXCR1/2, which serve as upstream mediators of NETosis and regulate immune‐mediated cytotoxicity against tumour cells, have demonstrated promising results in preclinical tumour models.^35^, ^134^, ^180^, ^181^, ^185^, ^186^, ^215^ Reparixin and SX‐682, specific inhibitors of CXCR1/2, have been reported to inhibit tumour infiltration of myeloid‐derived suppressor cells and reduce NETs extrusion in tumours, as demonstrated in murine BC, Lewis lung carcinoma and melanoma.^181^, ^185^ CXCR1/2 and IL‐8 inhibitors, as well as AZD5069 and Navarixin, have also been applied to pancreatic ductal adenocarcinoma, prostate cancer and diffuse large B‐cell, respectively lymphoma and other tumours.^35^, ^134^, ^180^, ^187^

##### PAD4 inhibitors

PAD4, a key enzyme involved in NETosis, can be effectively targeted to prevent histone H3 citrullination and subsequently inhibit NETosis. PAD4 inhibitor YW3‐56 has demonstrated the potential to enhance the radiosensitivity of NPC cells,^188^ while BMS‐P5 inhibits NETosis in vitro and decelerates the progression of multiple myeloma.^189^ Another PAD4 inhibitor GSK484^216^ could inhibit the radioresistant and invasive phenotypes of nasopharyngeal carcinoma cells in terms of tumour size, weight and volume, and it has been utilised in the treatment of ovarian cancer, HCC and melanoma. Similarly, BB‐Cl‐amidine is used to treat Ovarian cancer.^190^ Cl‐amidine is used to treat Ovarian cancer, BACA and melanoma.^27^, ^88^, ^190^ Chloramidine is used to treat pancreatic ductal adenocarcinoma.^193^ ZD‐E−1 M for the treatment of BACA and LC.^194^ Interestingly, PADI2Icariin can also be involved in the treatment of urothelial carcinoma as a target of NETosis.^195^ AntagomiR‐155,^217^ ZD‐E‐1,^194^ GSK199, GSK121 and GSK215^218^ have been shown to target PAD4 to inhibit NETosis, but their effectiveness in tumour therapy remains unexplored. Unfortunately, there is currently no approved pharmaceutical targeting PAD4 for therapeutic use in humans.

##### Autophagy inhibitors

To the extent that autophagy and NETosis can co‐occur and interact with each other, the utilisation of autophagy inhibitors has demonstrated efficacy in blocking NETosis, thereby impeding tumour progression.^10^, ^219^ Inhibition of autophagy with chloroquine has been reported to reverse the propensity for NETosis in vitro. It also exhibits enhanced suppression of NETs within the TME in murine models of pancreatic cancer. Additionally, chloroquine can reduce the hypercoagulability associated with tumours.^143^, ^220^ Low molecular weight heparin (LMWH) has demonstrated the ability to inhibit autophagy induction in activated neutrophils and the formation of NETs; however, its potential in tumour prevention and treatment remains unexplored.^219^

##### Antioxidants

NADPH oxidase‐mediated ROS generation is another important event of NETosis. Kaempferol reportedly inhibits the occurrence of lung metastases in breast cancer by targeting NETosis by participating in NADPH/ROS‐NETs signalling.^197^ Similarly, pepducin inhibits NETosis for pancreatic cancer.^187^ The NADPH inhibitor DPI is similarly involved in improving CRC.^198^ However, there are some ROS inhibitors that have been shown to inhibit NETosis in infectious diseases and autoimmune diseases to improve the disease, but whether they have an effect in tumours remains to be further studied. Specifically, Zingerone^221^ and octyl gallate^222^ can reduce the accumulation of ROS, inhibit NETosis and slow down the progression of sepsis. Tetramethylpyrazine (TMP) can prevent the complications of liver ischemia after liver transplantation.^223^ Gingerol^199^ can reduce systemic lupus erythematosus, antiphospholipid syndrome (APS) and NETosis. Metformin can inhibit mitochondrial ROS, deactivate the protein kinase C (PKC)‐NADPH oxidase (NOX) pathway to reduce NETosis, and has anti‐diabetic effects.^206^ In summary, the investigation of ROS inhibitors for the inhibition of NETosis in infectious diseases and autoimmune diseases has been extensively explored; however, their potential application in tumour therapies associated with NETs is currently underexplored.

##### GSDMD inhibitors

Recently, inhibitors targeting GSDMD—a key effector involved in cellular pyroptosis, NETosis and apoptosis^224^, ^225^—have demonstrated potential applications in anti‐NET oncology therapeutics by interfering with the N receptor to inhibit GSDMD. Ivermectin, a widely used antiparasitic drug, has also been found to inhibit NETosis to treat melanoma cancer metastases.^134^ Disulfiram (DSF), an FDA‐approved drug widely recognised for its potent anti‐inflammatory and anti‐cancer effects capable of influencing NETosis,^226^ has gained significant attention. Additionally, other GSDMD inhibitors like LDC7559^227^ and GSK598809 possess similar potential to impact NETosis thereby offering therapeutic options for this disease. Nevertheless, all these drugs are still in the early stages of research requiring extensive clinical trials to assess their safety profile as well as efficacy.

##### Other compounds

Given the number of factors involved in the formation of NETs, there are numerous drugs that inhibit NETosis for disease treatment. Acetylsalicylic acid (also known as aspirin), a non‐steroidal anti‐inflammatory drug that inhibits thromboxane A2, can inhibit NF‐κB and has been shown to mediate NETosis along with BAY‐11‐7082 and R0106‐9920.^228^ In BRCA, patients who take aspirin regularly have a significantly lower risk of mortality, recurrence and metastasis, leading to a better prognosis.^229^ When using aspirin for treatment, it is important to be mindful of potential side effects like gastric ulcers and compromised immune function.^206^ Thromboregulin can degrade HMGB1 and inhibit NETosis, thereby preventing the metastasis of pancreatic cancer to the liver.^120^ Antithrombin III is administered in the early stages of endotoxemia and reduces the NETosis, thereby improving patient survival.^200^ The TGF‐β signalling inhibitor LY2157299 can reduce the role of NETs in promoting GC cell proliferation, invasion, migration and eEMT,^201^ and SB525334 inhibition of TGF‐β 1 receptor may be used to treat pancreatic cancer.^202^ Compound AZD7986 targeting CTSC prevents lung metastasis of BRCA.^39^ Blockade of BCAM‐1 inhibits NETs‐induced cancer‐associated thromboembolism in pancreatic cancer.^203^ PGE1 can inhibit NETosis and delay pancreatic cancer.^205^ IL‐17 antibodies have been shown to inhibit NETosis in LC and pancreatic cancer to ameliorate cancer.^29^ Similarly, CEACAM1 monoclonal antibody has been confirmed to improve CRC.^164^ Integrin α5β1 inhibitor ATN‐161, DDR1 closely related to CXCL5 and KRAS mutation inhibitors have been shown to improve pancreatic ductal adenocarcinoma and pancreatic cancer and CRC.^41^, ^52^, ^198^

Additionally, the immuno‐receptor tyrosine activation motif coupled immunoglobulin‐like platelet receptor glycoprotein VI plays a crucial role in neutrophil recruitment and NETosis in experimental acute lung injury (ALI),^230^ and its inhibition may offer a promising approach to mitigate acute lung inflammation triggered by NETs in cancer‐associated ALI. Furthermore, cyclosporine A,^231^ activated protein C,^100^ PA‐dPEG24,^206^ SIRT3 agonists and magnolol^232^ can inhibit NETosis, which could be explored for targeted NETs treatment in cancer research.

Chemical inhibitors are often associated with off‐target effects. To mitigate this potential risk, researchers have increasingly focussed on employing gene editing techniques to achieve more precise targeting of NETosis. As early as 2010, Papayannopoulos et al. demonstrated that knocking out NE in mice significantly impaired NETs formation.^233^ Although this study was not directly cancer‐focussed, it provided a foundational understanding for subsequent investigations into the roles of NETs in cancer. Lulla et al. further used PyMT breast cancer models (NE+/+ and NE−/−), revealing that genetic ablation of NE significantly reduced lung metastasis and improved metastasis‐free survival.^138^, ^234^ In addition to knocking out NE, studies have indicated that both reduced NET formation and tumour growth occur in PAD4‐knockout Lewis LC and pancreatic cancer mouse models.^3^, ^4^, ^138^, ^142^ Additionally, knockout of PAD4 has been studied to inhibit the lung metastasis in breast cancer mouse model.^197^ These in vivo findings not only verify the regulatory function of NETs in tumours, but also further indicate that gene editing techniques targeting the key divers of NETosis may represent a highly promising approach for tumour therapy.

#### 5.3.2 Disruption of NETs structure

##### DNase‐I

DNase‐I, an endonuclease that selectively cleaves the phosphodiester bond in DNA, act as cutter to destroy the DNA scaffolds of NETs and lead to the loss of reticulation.^235^ Existing studies have shown that administration of DNase‐I via intravenous or intraperitoneal injections effectively reduces the levels of circulating and tumour‐infiltrating NETs, thereby non‐specifically attenuating metastatic features associated with matrix attachment, migration and invasion in multi‐tumour models.^236^ This DNase‐I treatment exhibits potent antimetastatic activity and decreases the likelihood of cancer recurrence and venous thrombosis following first‐line therapies such as radical surgery.^96^, ^237^ Notably, it has been applied to Phase I clinical trials in patients with head and neck cancer.^238^ Moreover, recombinant DNase‐I (such as, Pulmozyme) has been developed and is being investigated in related cancer treatments.^239^

Additionally, the incorporation of NETs‐targeted inhibitors onto vectors holds promise for augmenting the therapeutic efficacy of disease treatment. To improve the stability of DNase‐I, Hosseinnejad et al. conjugated DNase‐I with microgels synthesised from highly hydrophilic N‐(2‐hydroxypropyl) methacrylamide and zwitterionic carboxy betaine methacrylamide; this biomixing platform exhibited superior efficiency in NETs digestion compared to free DNase and also reduce NETs‐mediated inflammation and microthrombosis.^240^ Similarly, nanoparticles and adenovirus genes have been employed as carriers for DNase I in numerous studies to address the issue of its limited biological half‐life.^88^, ^241^ Yu et al. developed gold nanosystems loaded with RGD peptides and PAD4 inhibitors for combined chemo‐photothermal treatments, which significantly augmented the cytotoxicity against cells and demonstrated superior in vitro anti‐metastatic and invasive capabilities compared to individual therapies targeting either RGD or PAD4 inhibitors alone. Moreover, this approach effectively alleviated the leakage effect and biotoxicity associated with PAD4 inhibitors, thereby preventing lung metastasis while enhancing biosafety.^242^ Sun et al. developed a hybrid nanoparticle composed of DNase I and gold (DNase I@Au) to enhance radiotherapy efficacy by precisely eliminating NETs while attenuating lung metastasis in BACA.^207^ Yin et al. developed a smart nanocarrier consisting of a paclitaxel (PTX) prodrug nanoparticle core and a Tat peptide‐coupled DNase I shell of poly(l‐lysine) (PLL) coupled with MMP‐9 to modulate tumour‐associated NETs and enhance the inhibition of malignant tumour growth and distant metastasis.^208^ Chen J, et al., designed nanoplatforms with a broad‐spectrum photoactive plasmonic gold blackbody (AuPB) core and mesoporous dopamine (mPDA) shells to eliminate NETs‐mediated trapping of circulating tumour cells and hence tumour metastasis.^209^

##### Granin inhibitors

Proteins attached to the NETs structure are additional crucial components of NETs and have been receiving increasing attention as potential therapeutic targets, particularly with regard to NE and MPO. Sivelestat, a small molecule inhibitor targeting NE, has been shown to significantly reduce the growth of xenograft tumours and delay the progression of prostate and CRC.^85^, ^100^ It has been approved in Japan for the treatment of ALI associated with systemic inflammatory response syndrome.^211^ The NE inhibitor ONO‐5046 can inhibit the growth of LC.^81^ The serine protease inhibitor elafin counteracted the mitogenic effect of NE in the G0 phase of mammary epithelial cells and reduced the growth of BRCA.^213^ The therapeutic potential of MPO inhibitors is currently under investigation in a range of diseases, including systemic lupus erythematosus, asthma, tuberculosis, irritable bowel disease and depression.^243^, ^244^, ^245^ For example, AZM198 and PF‐1355 have demonstrated the ability to inhibit MPO activity and break the ring structure of NETs, thereby preventing immune complex vasculitis and renal injury.^243^ In conclusion, their application in tumour treatment is highly anticipated.

#### 5.3.3 Synergistic therapy

The aforementioned NETs‐target approaches can be synergistically applied for tumour treatment. For example, in a phase II clinical trial conducted on patients with advanced solid tumours, Navarixin (a CXCR1/2 antagonist) was used in combination with pembrolizumab to inhibit NETs, resulting in a significant delay in disease progression.^180^ Yang et al. proposed that the combination of DNase‐I with anti‐inflammatory drugs (such as aspirin or hydroxychloroquine) effectively attenuated HCC metastasis in mice model.^113^ Similarly, DNase I conjugated to PD‐1 antibody was used to improve CRC.^214^ Zhang et al. reported that inhibition of PAD4 synergised with PD‐1 blockade to dramatically reduce the tumour growth of pancreatic tumour.^29^

When synergistically applying therapeutic methods targeting NETs, some key factors warrant attention.^246^, ^247^, ^248^ For instance, concerning the efficacy evaluation criteria for combination strategies, should a single endpoint such as progression‐free survival or objective response rate be adopted, or should a composite endpoint system be utilised? Moreover, traditional efficacy evaluation criteria for solid tumours primarily assess therapeutic effects based on changes in tumour size. This may not be applicable to the targeted therapies of NETs. It is essential to incorporate the detection of NETs‐related markers in circulation and immune‐related indicators and so forth. This is because the biological effects of NETs‐targeted therapies are more prominently reflected in inhibiting the remodelling of the metastatic microenvironment or blocking the capture of circulating tumour cells. Furthermore, refining patient stratification criteria is crucial for the synergistic therapies.^249^ Differences exist in the levels of NETs within the TME among patients, the extent of tumour cells dependence on NETs, and the responsiveness of the immune system to NETs‐related signals. Based on these differences, along with considering clinical characteristics and immune status, precise patient stratification—such as dividing patients into high‐invasion group and low‐invasion group of NETs—can help identify populations most likely to benefit from targeted synergistic therapies.

Equally important is that different targeted drugs may lead to the accumulation of toxicity through shared or complementary mechanisms.^250^ For example, chemotherapy drugs often cause bone marrow suppression and decrease neutrophil counts,^251^ whereas NETs‐targeted therapies may impair neutrophils function. The combination of the two could exacerbate immunosuppression and elevate the risk of infection. In summary, research on NETs‐targeted synergistic therapies require comprehensive consideration of translational medicine issues, including the selection of efficacy evaluation criteria, patient stratification criteria and overlapping toxicity risks. Through in‐depth investigation on these aspects, promoting the translation of NETs‐targeted therapies from laboratory to clinical application becomes highly significant. Such efforts are expected to yield more scientific and effective combination treatment plans, ultimately enhancing therapeutic outcomes for cancer patients.

## 6 REMAINING CHALLENGES AND FUTURE DIRECTIONS

As our insights into the roles of NETs in cancer are expanding at an unprecedented rate, their presence has been identified not only in various solid tumours, but also in the peripheral circulation associated with these malignancies. Compared to the healthy control group, the levels of NETs in patients with solid tumours such as CRC, GC, LC and BRCA have significantly increased. This observation raises a critical question: Are the levels of NETs consistent across various types of cancer? To address this question, it is primarily necessary to determine how to quantify the levels of NETs in different populations. Some scholars utilised the transcriptome sequencing data of The Cancer Genome Atlas pan‐cancer primary focus to construct a NETs scoring system based on genes associated with NETosis. The results demonstrated that tumours originating from the brain and gastrointestinal tract generally exhibited higher NETs scores, whereas those from secretory glands presented lower NETs scores.^171^, ^252^ This research indicates that the distinct origins, molecular biological features and TME disparities of various tumours might render NETs with considerable heterogeneity and complexity. Of note, the scoring system based on the RNA levels of NETosis‐related genes may not precisely represent the actual levels of NETs formed in tumours. Actually, there is a diverse range of NETs‐related markers, including NETs‐DNA, CitH3, NE/NE‐DNA and MPO/MPO‐DNA, and so forth. Quantification using different markers may cause a certain degree of deviation in the levels of NETs. Even for the same marker, its detection results may vary due to the differences in detection technology. Hence, establishing a standardised quantitative detection protocol for NETs is a key challenge in achieving their clinical application value.

Herein, it is noteworthy that the rapidly advancing multi‐omics technologies hold revolutionary potential for understanding how NETs influence tumour progression. Currently, traditional research methods are limited by single‐dimensional data and thus fail to accurately capture the dynamic changes of NETs within the TME. In contrast, spatial transcriptomics enables the generation of high‐resolution molecular maps of tumours, while single‐cell proteomics can allows for in‐depth analysis of proteins expression and modifications in tumours. These technologies will facilitate a comprehensive dissection of the protein spectra associated with NETs formation and function at the single‐cell level, as well as an exploration of the spatiotemporal‐specific mechanisms underlying NETs‐mediated tumourigenesis and metastasis. Nonetheless, single‐cell omics and spatial multi‐omics face challenges in data integration and interpretation, such as effectively consolidating the complex datasets generated by diverse technologies. These are urgent problems that need to be solved.

The application of NETs in clinical tumour management lies in their potential as tumour biomarkers and therapeutic targets. To realise the clinical transformation of NETs as tumour markers, optimisation is requisite in two respects. Firstly, considering the insufficient specificity in single‐marker detection, it is advisable to adopt a multi‐marker combined detection strategy. For example, integrating multiple NETs‐specific markers with existing clinical tumour markers may help enhance the result's specificity. Secondly, the development of specific antibodies against specific epitopes of NETs and improved detection techniques are expected to overcome the specificity and sensitivity bottleneck of existing detection methods. It is important to highlight that, as of now, no reference range for NETs has been established based on data from large‐scale healthy populations. Most existing studies have primarily focussed on comparing the NETs levels between tumour patients and healthy controls within single‐centre cohorts. To advance the clinical application of NETs as biomarkers, it is essential to establishing a standardised and universally applicable definition of ‘normal’ NETs levels. There are also some challenges in targeting tumour therapy strategies for NETs, such as the potential off‐target effects of inhibitors, the risk of compromising host defence and the presence of drug resistance. Furthermore, in the synergistic application of NETs‐targeting approaches, additional challenges involve establishing appropriate efficacy evaluation criteria, refining patient stratification standards and effectively managing overlapping toxicity risks. At the same time, although NETs inhibitors can suppress tumour development in preclinical models, they still have a long way to go before clinical translation. These will need to be validated in future clinical trials.

## AUTHOR CONTRIBUTIONS

Peilong Li and Lutao Du conceived and designed the research; Yifan Wang and Kangjie Yang performed the literature search, drafted the manuscript and created figures; Juan Li and Chuanxin Wang revised the manuscript and figures. All the authors read and approved the final manuscript.

## CONFLICT OF INTEREST STATEMENT

The authors declare no conflicts of interest.

## ETHICS STATEMENT

Ethical approval is not applicable to this study.

## Data Availability

Data sharing not applicable to this article as no datasets were generated or analysed during the current study.
